# Tumor Necrosis Factor-Like Weak Inducer of Apoptosis Promotes Hepatic Stellate Cells Migration via Canonical NF-κB/MMP9 Pathway

**DOI:** 10.1371/journal.pone.0167658

**Published:** 2016-12-01

**Authors:** Mingcui Xu, Feng Zhang, Aixiu Wang, Chen Wang, Yu Cao, Ming Zhang, Mingming Zhang, Min Su, Xiaoping Zou, Guifang Xu, Yuzheng Zhuge

**Affiliations:** 1 Department of Gastroenterology, Affiliated Drum Tower Clinical Medical School of Nanjing Medical University, Nanjing, Jiangsu, China; 2 Department of Gastroenterology, Drum Tower Hospital, Affiliated to Medical School of Nanjing University, Nanjing, Jiangsu, China; University of Louisville School of Medicine, UNITED STATES

## Abstract

In the liver, the signal and function of tumor necrosis factor-like weak inducer of apoptosis (TWEAK) have mainly been assessed in association with liver regeneration.

However, the effects of TWEAK on liver fibrosis have not been fully elucidated. To investigate the effects of TWEAK on human hepatic stellate cells (HSCs) and to explore the relevant potential mechanisms, human HSCs line—LX-2 were cultured with TWEAK. Cell migration was detected by transwell assay; cell viability was evaluated by Cell Counting Kit-8; the expression of MMP1, MMP2, MMP3, MMP7, MMP8, MMP9, MMP10, MMP11, MMP12, MMP13 gene was identified by quantitative real-time polymerase chain reaction and western blotting; the activity of matrix metalloproteinases (MMPs) was tested by enzyme-linked immuno sorbent assay; small interfering RNA transfection was applied for depletion of MMP9 and p65. The result of transwell assay revealed that TWEAK promoted LX-2 migration. Subsequently, our data testified that the expression and activity of MMP9 was induced by TWEAK in LX-2 cells, which enhanced the migration. Furthermore, our findings showed that TWEAK upregulated the phosphorylation of IκBα and p65 protein to increase MMP9 expression in LX-2 cells. Meanwhile, the alpha-smooth muscle actin, vimentin and desmin expression were upregulated following TWEAK treatment. The results in the present study revealed that TWEAK promotes HSCs migration via canonical NF-κB/MMP9 pathway, which possibly provides a molecular basis targeting TWEAK for the therapy of liver fibrosis.

## Introduction

Liver fibrosis is an outcome that caused by almost all chronic hepatic diseases, such as viral hepatitis, alcoholic or nonalcoholic steatohepatitis, drug induced liver injury[1]. Liver fibrosis can further progress into cirrhosis, the severe complications of which bring poor prognosis. Thus, it is clearly important to explore the intricate mechanisms of liver fibrosis and to develop targeted therapies. The activation of hepatic stellate cells (HSCs), which transdifferentiates into myofibroblasts, has been known as a crucial pathogenic step in the development of liver fibrosis[1–4]. Myofibroblasts are not present in healthy liver, whereas they are discovered in chronic injured liver. Myofibroblasts are considered to be a key regulator of fibrogenesis owing to their enhanced migration[5, 6], contractility and producing excessive extracellular matrix (ECM)[7, 8]. In our study, the activated human HSCs line—LX-2 was used in our research. Although LX-2 cells are different from the primary HSCs, they have the characteristics of activated HSCs[9,10]. Recent studies have shown that the matrix metalloproteinases (MMPs) are capable of degrading virtually any components of the ECM, which play a pivotal role in the migration of cells[11,12]. However, whether the enhanced migration of the activated HSCs was associated with MMPs has not been revealed.

Tumor necrosis factor-like weak inducer of apoptosis (TWEAK) is a member of tumor necrosis factor ligand superfamily, which is a kind of type Ⅱ transmembrane protein and can be cleaved proteolytically to generate a soluble protein. TWEAK functions physiologically after acute injury and pathologically in chronic inflammatory disease settings[13–15]. It has been reported that TWEAK is involved in numerous cellular processes including cell survival, proliferation, differentiation, migration and apoptosis[16]. In the liver, the signal and function of TWEAK have mainly been explored in liver regeneration[17]. It has been reported that the dominant function of TWEAK is to induce liver progenitor cells expansion[18]. However, the investigation of TWEAK on liver fibrosis is limited. Interactions between TWEAK and its receptor, fibroblast growth factor-inducible 14 (Fn14) have been reported to regulate fibrosis in several organs including the heart, kidney, colon and muscle[19]. Whereas, the effects of TWEAK on liver fibrosis and HSCs has not been fully demonstrated.

The aim of this study was to investigate the effects of TWEAK on HSCs, and to explore the underlying mechanisms. We focused on the MMPs expression and the marker of myofibroblasts expression to indicate that TWEAK promoted HSCs migration via regulating MMPs expression.

## Materials and Methods

### Materials and chemicals

LX-2 cells[10] (#SCC064) were purchased from Merk Millipore, USA in December, 2015. Recombinant Human TWEAK/TNFSF12, 25 ug (1090-TW) was obtained from R&D system. BCA Protein Assay Kit was supplied by Keygen Biotech (Nanjing, China). Cell Culture Inserts were obtained from BD Biosciences, USA. Transwell chambers (pore size 8um) were purchased from BD Biosciences, USA. Cell Counting Kit-8 (CCK-8) kit was purchased from DOJINDO Laboratories, Japan. MMP9 and p65 siRNA were acquired from RiboBio (Guangdong,China). The PrimeScript RT Master Mix and SYBR Premix Ex Taq reagents for qRT-PCR were gained from Takara Biotechnology, Japan. IκBα (ab32518), MMP7 (ab205525), MMP8 (ab81286), MMP9 (ab137867), MMP13 (ab51072), alpha-smooth muscle actin (α-SMA) (ab124964), desmin (ab32362), vimentin (ab92547) monoclonal antibodies were purchased from Abcam Company, UK. MMP9 Elisa kit (ab100610) was obtained from Abcam Company, UK. MMP7 (ELH-MMP7-1), MMP8 (ELH-MMP8-1), MMP13 (ELH-MMP13-1) Elisa kits were supplied by RayBiotech, USA. p-IκBα (Phospho-IκBα Ser32/36, 9246s), p65 (NF-κB p65, 4764s), p-p65 (Phospho-NF-κB p65 Ser536, 3033p) monoclonal antibodies were obtained from CST, USA. DMEM and fetal bovine serum were obtained from Biological Industries, Israel. 0.25% trypsin was acquired from Gibco, USA.

### Cell culture

LX-2 cells were maintained in DMEM medium with 10% fetal bovine serum at 37°C in a humid incubator supplemented with 5% CO_2_. 80% confluent cells were used in the experiments.

### TWEAK treatment

The lyophilized powder of TWEAK was dissolved with PBS and stored at -80°C; the dissolved TWEAK was diluted with PBS for the next experiments. The cells were treated with 40ng/ml and 100ng/ml TWEAK for 24h, and the control group was treated with vehicles (PBS). The specific concentration of TWEAK was based on our CCK-8 assay.

### Transwell assay

The transwell assay was performed using a 8 um pore size Transwell system. In brief, the chambers set on 24 well plates. LX-2 cells suspended in 500ul of DMEM were seeded in the upper chamber at a density of 3×10^4^ cells/well. 750ul of DMEM containing 15% fetal bovine serum were added into the lower chamber. After 24 h incubation, the cells in the upper chamber were removed, and the cells that migrated through the membrane to the underside were fixed with methanol for 15 min and then stained with 0.5% crystal violet for 15 min. Cell number in five separate fields were counted using light microscopy at 200× magnification. The assay was repeated three times independently.

### Cell viability assay

Cell viability was observed by CCK-8 assay. The cells were seeded into 96-well plates at a density of 4×10^3^ cells per well and incubated for 24 h at 37°C. After treatment with a range concentrations (0, 20, 40, 80, 100 ng/ml) of TWEAK or PBS for indicated time, 10 ul CCK-8 was added into per well. Plates were cultured for 1.5 h at 37°C. The absorbance of samples (450 nm) was investigated by a scanning multiwell spectrophotometer. Cell viability was calculated by the following formula: relative cell viability = (absorbance450nm of treated group−absorbance450nm of blank)/(absorbance450nm of control group−absorbance450nm of blank). All experiments were done in triplicate and repeated three times independently.

### Quantitative real-time polymerase chain reaction (QRT-PCR)

Total RNA in the cells was extracted using the Trizol Reagent (Invitrogen Life Technologies, USA) and subsequently reverse transcribed using the PrimeScript RT Master Mix according to the manufacturer’s instructions. QRT-PCR was done with the 7500 Real-time PCR System (Applied Biosystems) using SYBR Premix Ex Taq reagents. PCR cycling conditions were: 40 cycles of 5 s at 95°C,32–34 s at 60°C. All data were normalized to the human β-actin. Fold-induction was calculated using the formula 2^-(ΔΔCt)^. Data represented was based on three independent experiments. The PCR primers were bought from Sangon Biotech, Shanghai, China. Primer sequences are shown in Table 1.

**Table 1:** List of primer sequences.

Target gene	Forward primer (5'→3')	Reverse primer (5'→3')
MMP1	ACGAATTTGCCGACAGAGAT	GGAAGCCAAAGGAGCTGTAG
MMP2	CAAGGACCGGTTCATTTGGC	GGCCTCGTATACCGCATCAA
MMP3	GTCCCTCTATGGACCTCCCC	AGGGATTTGCGCCAAAAGTG
MMP7	GTCTCTGGACGGCAGCTATG	GATAGTCCTGAGCCTGTTCCC
MMP8	ACCAAAGAGATCACGGTGACA	TGGTCCATGTTTCTTCGGCA
MMP9	GATCATTCCTCAGTGCCGGA	TTCAGGGCGAGGACCATAGA
MMP10	GACAGAAGATGCATCAGGCAC	CATCTTGCGAAAGGCGGAAC
MMP11	AAGAGGTTCGTGCTTTCTGG	CGTCACATCGCTCCATACC
MMP12	ACACATTCAGGAGGCACAAAC	GTCATCAGCAGAGAGGCGAA
MMP13	TGCAGAGCGCTACCTGAGAT	AGACTGCATTTCTCGGAGCC
α-SMA	GTTCCGCTCCTCTCTCCAAC	ACGCTGGAGGACTTGCTTTT
vimentin	CGGGAGAAATTGCAGGAGGA	AAGGTCAAGACGTGCCAGAG
desmin	CCATACCAAGAAGACGGTGA	GAGGACTGAGGCTGGGTGT
β-actin	AGCGAGCATCCCCCAAAGTT	GGGCACGAAGGCTCATCATT

### Western blotting

Cells were lysed in RIPA buffer (30 mM Tris, pH 7.5, 150 mM sodium chloride, 1 mM phenylmethylsulfonyl fluoride, 1 mM sodium orthovanadate, 1% Nonidet P-40, 10% glycerol, and phosphatase and protease inhibitors). BCA kit was applied for protein quantification. Then the protein was denaturated in boiling water for 10 minutes. The protein was separated by SDS-PAGE and transferred to polyvinylidene fluoride membranes. The membranes were blocked by 5% non-fat dry milk in Tris buffered saline containing 0.1% Tween-20 for 2 h at room temperature. Then the membranes were incubated with primary antibodies according to the instructions overnight at 4°C followed by probed by the HRP-conjugated appropriate secondary antibodies (1:5000 dilution). The blots were detected by CCD camera (Tanon, Shanghai, China) with enhanced chemiluminescence (Millipore, USA). Data are representative of at least three independent experiments.

### Enzyme-linked immuno sorbent assay (Elisa)

The LX-2 cells were seeded in 6 wells plates at a density of 1.5 × 10^5^ cells/well overnight at 37°C. After treatment with TWEAK at indicated concentrations or PBS as control for 24h, the cell culture medium was collected. The qualification of the activated MMPs in the medium was measured according to the manufacturer’s instructions. All experiments were done in triplicate and repeated three times independently.

### Small interfering RNA (siRNA) transfection

SiRNA duplexes used in this study were purchased from RiboBio with the following sequences MMP9 (5’-GTACCGCTATGGTTACACT-3’), p65 (5’-CTTCCAAGTTCCTATAGAA-3’) and negative control (5’-TTCTCCGAACGTGTCACGTTT-3’). LX-2 cells were seeded in six-well plates and transfected at 60% confluency with siRNA duplexes against human MMP9 (20 nM), p65 (20 nM), or control siRNA (20 nM) respectively, by Lipofectamine RNAiMAX reagent (Invitrogen) according to the manufacturer’s protocol. At 48 h post-transfection, cells were cultured with TWEAK (100ng/ml) or PBS for 24 h before harvest for the next experiment.

### Phalloidin staining

Cells grown in six well plates were fixed in 4% paraformaldehyde for 15 min and permeabilized using 0.5% Triton X-100/PBS for 20 min at room temperature. The cells were blocked with PBS supplemented with 2% bovine serum albumin for 1 h at room temperature. Filamentous actins were stained with FITC-labeled Phalloidin (5 ug/ml, Sigma-Aldrich) and nucleus were stained with DAPI (2 ug/ml) for 20 min at room temperature. The fluorescence microscopy (Olympus, Japan) was used to investigated the morphology of the cells. The assay was repeated for three times.

### Statistical analysis

All the data presented by mean±SD of three independent experiments were analysed by SPSS 20.0. Unpaired student’s t test was applied for two groups comparison. One-way analysis of variance was used for multiple groups comparison. *P<*0.05 was considered statistically significant.

## Results

### TWEAK promoted LX-2 cells migration

**Fig 1:**
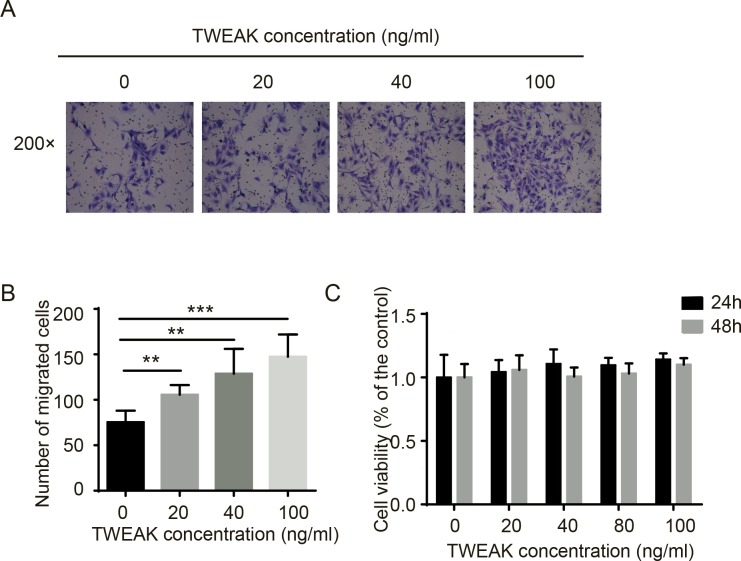
LX-2 cells migration was enhanced by TWEAK. (A) LX-2 cells were treated with vehicle (PBS) or 20 ng/ml, 40 ng/ml and 100 ng/ml TWEAK for 24 h, then the transwell assay was performed. The data shown here are from three independent experiments with similar results. Original magnification 200×. (B) The number of migrated cells were displayed as histogram, compared with the control group. The data were displayed as the mean value of cells in five fields based on three independent experiments. (C) LX-2 cells were treated with vehicle (PBS) or vary concentrations of TWEAK for 24 h or 48 h and assayed by CCK-8. Results are expressed as the mean ± SD of three independent experiments. **P<0.05, ***P<0.01 when compared with the control group.

LX-2 cells were treated with 20 ng/ml, 40 ng/ml and 100 ng/ml TWEAK for 24 h, the transwell assay revealed that the number of the migrated cells was significantly increased in TWEAK-treated groups compared to the control group (t = -4.057, *P*<0.05, t = -3.199, *P<*0.05 and t = -4.319, *P<*0.01 respectively) (Fig 1A and 1B). In addition, LX-2 cells were incubated in medium with a range concentrations (0, 20, 40, 80, 100 ng/ml) of TWEAK for 24 h or 48 h. The cell viability was evaluated by CCK-8 assay. As shown in Fig 1C, different concentrations of TWEAK didn’t notably affect LX-2 cells viability compared to the control group.

### TWEAK significantly upregulated the expression of MMP7, MMP8, MMP9, MMP13 and the activity of MMP9 in LX-2 cells

**Fig 2:**
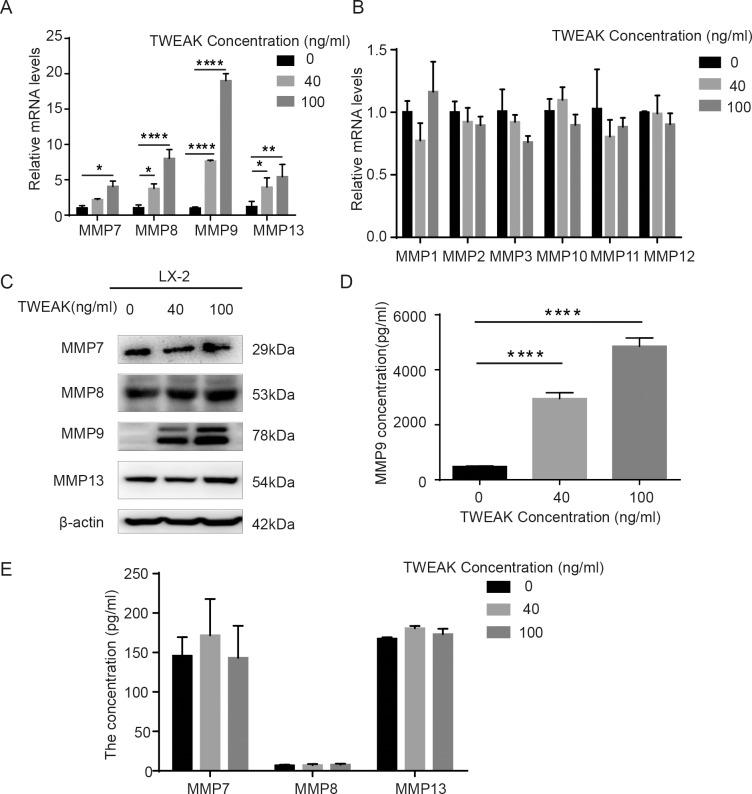
TWEAK significantly increased the expression of MMP7, MMP8, MMP9, MMP13 and the activity of MMP9 in LX-2 cells. (A) The mRNA expression of MMP7, MMP8, MMP9 and MMP13 were measured by qRT-PCR in LX-2 cells treated with 40 ng/ml and 100 ng/ml TWEAK for 24 h. β-actin served as an internal control. (B) The mRNA of MMP1, MMP2, MMP3, MMP10, MMP11 and MMP12 was examined by qRT-PCR in LX-2 cells treated with 40 ng/ml and 100 ng/ml TWEAK for 24 h. β-actin served as an internal control. (C) Western blotting to examine the expression of MMP7, MMP8, MMP9 and MMP13 in LX-2 cells treated with 40 ng/ml and 100 ng/ml TWEAK for 24 h. β-actin was used as a loading control. (D) Activated MMP9 expression in LX-2 cells culture medium was investigated by Elisa after being treated with 40 ng/ml and 100 ng/ml TWEAK for 24 h. (E) Activated MMP7, MMP8 and MMP13 in LX-2 cells culture medium were tested by Elisa after being treated with 40 ng/ml and 100 ng/ml TWEAK for 24 h. Results are expressed as the mean ± SD of three independent experiments. *P<0.05, **P<0.01,****P<0.0001.

To explore the potential molecular mechanisms that TWEAK facilitated LX-2 cells migration, the expression of MMPs in LX-2 cells was detected. The result of qRT-PCR showed that the messenger RNA (mRNA) of MMP7, MMP8, MMP9, MMP13 was significantly increased by TWEAK (*P<*0.05) for 24 h treatment (Fig 2A), especially MMP9 got a rise of 7 fold in 40 ng/ml TWEAK (*P<*0.0001) and 20 fold in 100 ng/ml TWEAK (*P<*0.0001), while the mRNA of MMP1, MMP2, MMP3, MMP10, MMP11, MMP12 in LX-2 cells was not notably changed compared to the control group (Fig 2B). Next, western blotting was performed to detect the protein of MMP7, MMP8, MMP9, MMP13 in LX-2 cells. And the result revealed that only MMP9 protein in cell lysates was significantly upregulated in response to TWEAK (Fig 2C). As is known to us, MMPs are secreted to the extracellular environment to act as soluble proteins, which play roles of activity. Thus, the cell culture medium was collected to assess the activity of MMPs with Elisa. And the result demonstrated that TWEAK significantly increased the activity of MMP9 in the medium (*P<*0.0001) (Fig 2D) but not MMP7, MMP8 and MMP13 compared to the control group (Fig 2E).

### MMP9 knocking down attenuated LX-2 cells migration enhanced by TWEAK

**Fig 3:**
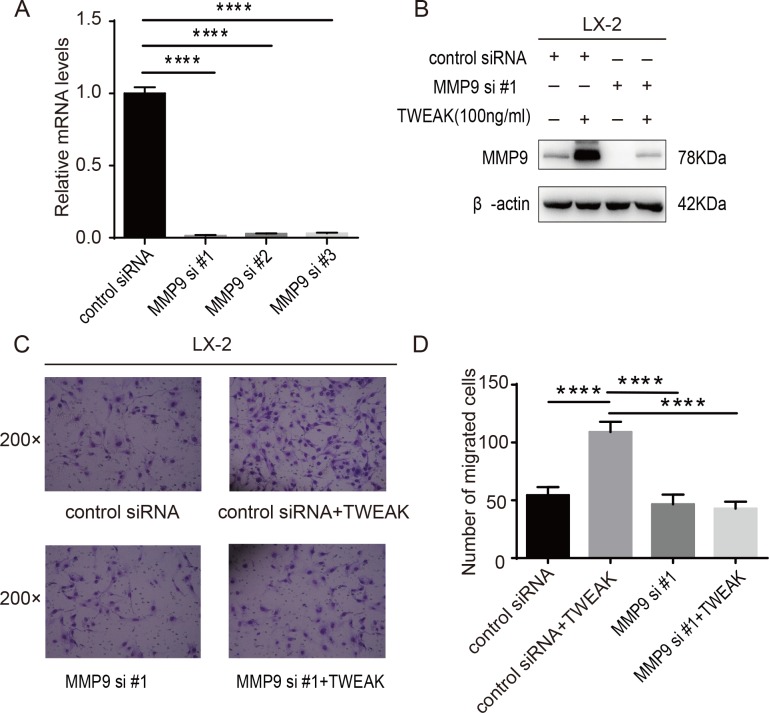
MMP9 knocking down inhibited the migration of LX-2 cells in response to TWEAK. (A) QRT-PCR were used to examine the expression of MMP9 in LX-2 cells transfected with control siRNA (20 nM) or siRNA specific for MMP9 (20 nM). β-actin served as an internal control. (B) LX-2 cells were transfected with control siRNA (20nM) or siRNA specific for MMP9 (20nM) for 48 h and further incubated with 100 ng/ml TWEAK for 24 h. The effect of MMP9 knocking down was analyzed by western blotting. (C) LX-2 cells were transfected with control siRNA or siRNA specific for MMP9 for 48 h and further incubated with 100 ng/ml TWEAK for 24 h. The migration was analysed by transwell assay. Original magnification 200×. (D) The number of migrated cells were displayed as histogram, data presented as mean ± SD are representative of three independent experiments when compared with other groups. ****P<0.0001.

To reveal whether TWEAK promoted LX-2 cells migration via upregulating MMP9 expression, MMP9-specific siRNA was used to knock down MMP9 gene expression. The effect of MMP9 knocking down was validated by qRT-PCR and western blotting (Fig 3A and 3B). As is shown in Fig 3C and 3D, knocking down of MMP9 expression significantly decreased the number of migrated cells enhanced by 100 ng/ml TWEAK for 24h treatment (*P<*0.0001), indicating that TWEAK strengthened LX-2 cells migration through increasing MMP9 expression.

### TWEAK activated canonical NF-κB pathway to upregulate MMP9 expression in LX-2 cells

**Fig 4:**
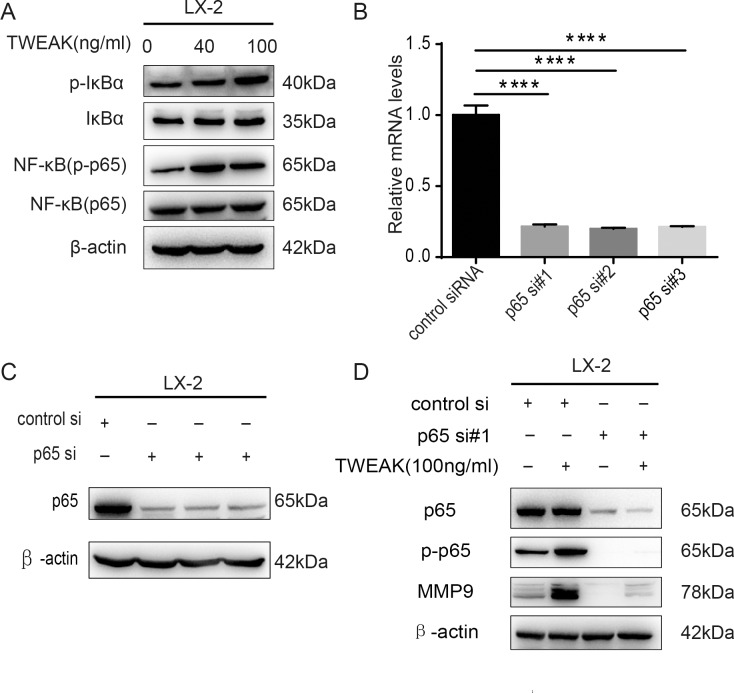
TWEAK induced canonical NF-κB pathway activation then augmented MMP9 expression in LX-2 cells. (A) Whole cell extracts were prepared and analyzed by western blotting for the p-IκBα, IκBα and p-p65, p65 in LX-2 cells treated with 40 ng/ml and 100 ng/ml TWEAK for 24 h. β-actin served as an internal control. (B) The expression of p65 in LX-2 cells transfected with scrambled RNA (20 nM) or siRNA specific for p65 (20 nM) was examined by qRT-PCR (B) and western blotting (C). β-actin served as an internal control. (D) LX-2 cells were transfected with control siRNA (20nM) or siRNA specific for p65 (20nM) for 48 h and further incubated with TWEAK (100 ng/ml) for 24 h. The protein of p65, p-p65 and MMP9 in cell lysates were measured by western blotting. β-actin was used as a loading control. All data are represented as mean ± SD of three independent experiments. ****P<0.0001.

To evaluate whether NF-κB activation was involved in the increased MMP9 expression in LX-2 cells treated with TWEAK, we detected IκBα and p65 protein (which were the components of canonical NF-κB pathway) in LX-2 cells exposed to 40 ng/ml and 100 ng/ml TWEAK for 24h. The western blotting showed that phosphorylation of IκBα and p65 protein was significantly increased by TWEAK, indicating a considerable activation of canonical NF-κB pathway (Fig 4A). Next, to study whether the activation of canonical NF-κB pathway enhanced MMP9 expression, the p65-specific siRNA was transfected into the LX-2 cells. QRT-PCR and western blotting were used to validated the efficacy of the depletion of p65 (Fig 4B and 4C). As is shown in Fig 4D, knocking down of p65 expression significantly decreased the MMP9 expression enhanced by 100 ng/ml TWEAK for 24 h treatment, indicating that TWEAK upregulated MMP9 through activating canonical NF-κB pathway.

### TWEAK increased the expression of α-SMA, vimentin, desmin in LX-2 cells and changed the cell morphology

**Fig 5:**
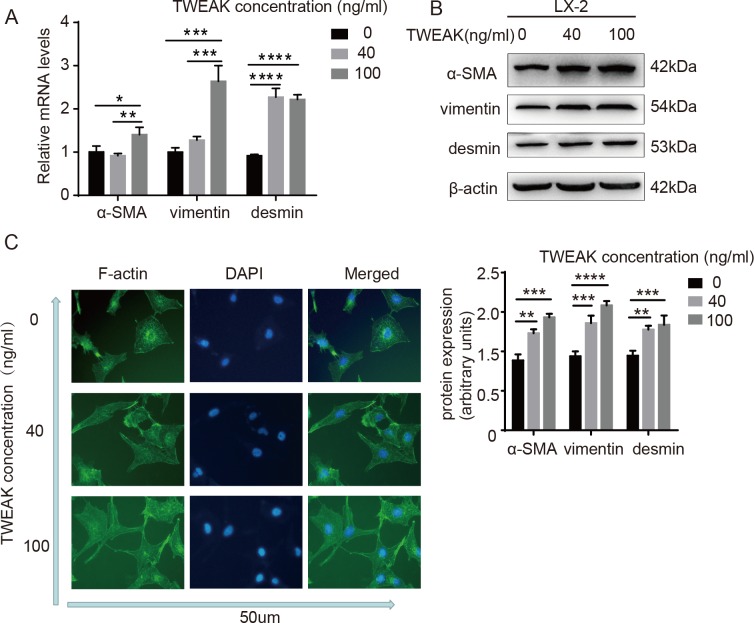
TWEAK significantly increased the expression of α-SMA, vimentin, desmin in LX-2 cells and changed the cell morphology. The mRNA (A) and protein (B) of α-SMA, vimentin, desmin in LX-2 cells treated with 40 ng/ml and 100 ng/ml TWEAK for 24 h were analyzed by qRT-PCR and western blotting. β-actin served as an internal control. Data are presented as mean ± SD of three independent experiments. (C) Phalloidin stained actin cytoskeleton (green) in LX-2 cells. The nuclear was stained by DAPI (blue). The data shown here are from three independent experiments with similar results. Scale bars, 50um. *P<0.05,**P<0.005,***P<0.0005,****P<0.0001.

Our results showed that TWEAK promoted LX-2 cells migration. Enhanced migration is one of the characters of myofibroblasts. The α-SMA, vimentin and desmin were considered as the marker of myofibroblasts. To assess whether TWEAK could increase the expression of myofibroblasts marker in LX-2 cells, the mRNA and protein of the myofibroblasts marker were tested. The results documented that α-SMA, vimentin and desmin expression were significantly upregulated by TWEAK treatment in LX-2 cells (Fig 5A and 5B). The cell morphology conversion was confirmed by visualizing actin cytoskeleton rearrangements by phalloidin staining (Fig 5C). It was obvious that a spindle-shape morphology and the presence of long surface parapodium of LX-2 cells were appeared with TWEAK treatment. The results indicated that TWEAK made LX-2 cells much more activated.

## Discussion

Here we have provided evidence that the effects of TWEAK on LX-2 cells. TWEAK facilitated LX-2 cells migration through increasing MMP9 expression. Furthermore, the potential mechanism was that TWEAK activated canonical NF-κB/MMP9 pathway to promote LX-2 cells migration. In addition, we also testified that TWEAK made LX-2 cells much more activated.

Previous studies revealed that TWEAK was involved in organ fibrosis. In an animal model of mammary tumorigenesis, endurance training prevents TWEAK mediated cardiac remodeling in cancer cachexia[20]. In kidney, TWEAK induces proximal tubular cells epithelial-mesenchymal transition, which starts tubulointerstitial damage and fibrosis[21]. However, the effects of TWEAK on liver fibrosis has not been fully accessed. Recently, Annika Wilhelm *et al*. indicated that TWEAK was upregulated in an animal model of acute and chronic liver injury, TWEAK knock-out mice presented with reduced liver fibrosis upon chronic CCl4 treatment[22]. These findings demonstrated that TWEAK promoted the progression of liver fibrosis. However, the potential mechanisms remain to be elucidated.

In the present study, we showed for the first time that TWEAK facilitated human HSCs line—LX-2 cells migration. It has been reported that the migration and invasiveness are associated with MMPs. In non-small cell lung cancer (NSCLC), Yu *et al*. suggested that the overexpression of MMP19 promotes migration and invasiveness in multiple NSCLC cell lines, and the upregulated MMP19 gene expression implies a poorer prognosis[23]. In hepatocellular carcinoma (HCC), Garcia-Irigoyen *et al*. documented that human HCC cells highly expression MMP10 has increased migratory capacity, which contributes HCC progression and metastasis[24]. Our research showed that TWEAK enhanced LX-2 cells migration, then qRT-PCR was applied to test the mRNA expression of MMPs, ranging from MMP1 to MMP13, in that their basal expression keep high levels in liver. The results showed that the mRNA of MMP7, MMP8, MMP9, MMP13 was increased in response to TWEAK treatment. However, only the protein expression and activity of MMP9 were significantly upregulated. It tells us that maybe post-transcriptional modification was involved in the MMP7, MMP8, MMP13 gene expression, whereas, this needs further studies. Furthermore, our data validated that the depletion of MMP9 gene expression significantly reduced the enhanced migration of LX-2 cells induced by TWEAK. It tells us that the strengthened migration was modulated by MMP9 gene in LX-2 cells.

MMP9 is a member of gelatinases. It isn’t present in naive liver and can be induced under different liver conditions[25]. MMP9 is a multifunctional protein, including mediating leukocyte traffic in hepatic ischemia and reperfusion injury[26], aggravating drug associated acute liver injury[27], regulating hepatic regeneration[28] and liver fibrosis[29]. It has been reported that MMP9 expression is observed in the early stages of liver fibrogenesis and MMP9 could activated latent transforming growth factor beta, a major profibrotic cytokine[30]. Ehling *et al*. documented that the pro-angiogenic gene vascular endothelial growth factor and MMP9 were upregulated by macrophages in injured livers to form new blood vessel, which may contribute to the progression of hepatic fibrosis[31]. Munch *et al*. revealed that MMP9 was associated with fibrosis and cardiac events in hypertrophic cardiomyopathy[32]. Our study reveals that TWEAK via increasing MMP9 expression facilitates HSCs migration. It tells us that MMP9 may play a role in the progression of liver fibrosis. Overall, the agents targeting MMP9 for the therapy of liver fibrosis is worthy to be explored.

Our study revealed that TWEAK upregulated MMP9 expression in LX-2 cells. Henaut *et al*. demonstrated that TWEAK favors calcification of vascular smooth muscle cells through activating canonical NF-κB pathway. Blockade of canonical NF-κB pathway reduced by 80% of TWEAK pro-calcific properties[33]. Pettersen *et al*. documented that TWEAK binding to Fn14 receptor has been shown to activate NF-κB signaling, which is important in cancer therapy resistance and tumorigenesis[34]. Our research for the first time verified that TWEAK activates canonical NF-κB pathway in LX-2 cells, which leads to an increase in MMP9 expression. In addition, our findings associated the upregulated MMP9 expression with the enhanced migration of LX-2 cells.

Enhanced migration and myofibroblasts phenotype are notable characters of activated HSCs[35]. Activated HSCs play a key role in liver fibrogenesis owing to their contractility phenotype and producing excessive ECM, which lead to portal hypertension and the stiffness of liver tissue. It indicates that TWEAK accelerated the progression of liver fibrosis. Moreover, our data revealed that 100 ng/ml TWEAK may have more potent effect on the progression of liver fibrosis than 40 ng/ml TWEAK. The transwell assay showed that the amount of the migrated cells in 100ng/ml TWEAK was more than 40 ng/ml TWEAK, although this didn’t achieve statistically significant (Fig 1B). Additionally, the qRT-PCR, western blotting and Elisa for MMP9 expression showed that 100 ng/ml TWEAK had higher level than 40ng/ml TWEAK (Fig 2A, 2C and 2D). Moreover, the mRNA and protein expression of myofibroblasts marker in LX-2 cells also indicated 100 ng/ml TWEAK presented higher levels when compared to 40 ng/ml TWEAK (Fig 5A and 5B).

## Conclusions

In conclusion, the present study elucidated that TWEAK promotes HSCs migration via canonical NF-κB/MMP9 pathway. All of these results indicated that TWEAK promoted HSCs activation. The obtained findings may provided a molecular basis for the treatment of liver fibrosis by targeting TWEAK.
